# Graphene-Based Electrode Materials for Neural Activity Detection

**DOI:** 10.3390/ma14206170

**Published:** 2021-10-18

**Authors:** Weichen Wei, Xuejiao Wang

**Affiliations:** 1 Materials Science and Engineering Program, University of California San Diego, La Jolla, CA 92093, USA; w4wei@eng.ucsd.edu; 2 Fujian Provincial University Engineering Research Center of Industrial Biocatalysis, College of Chemistry and Materials Science, Fujian Normal University, Fuzhou 350007, China

**Keywords:** neural sensors, graphene-based materials, flexible electronics, implantable electrodes

## Abstract

The neural electrode technique is a powerful tool for monitoring and regulating neural activity, which has a wide range of applications in basic neuroscience and the treatment of neurological diseases. Constructing a high-performance electrode–nerve interface is required for the long-term stable detection of neural signals by electrodes. However, conventional neural electrodes are mainly fabricated from rigid materials that do not match the mechanical properties of soft neural tissues, thus limiting the high-quality recording of neuroelectric signals. Meanwhile, graphene-based nanomaterials can form stable electrode–nerve interfaces due to their high conductivity, excellent flexibility, and biocompatibility. In this literature review, we describe various graphene-based electrodes and their potential application in neural activity detection. We also discuss the biological safety of graphene neural electrodes, related challenges, and their prospects.

## 1. Introduction

Neural electrodes provide an interface for the effective information transmission between the nervous system and external devices, which not only can be potentially used in fundamental neuroscience research, such as exploring the mechanisms of cognitive processes and the neural basis of sensory information processing, but also help find cures for many neurological diseases [1,2,3]. For example, recording brain activity by neural electrodes may identify the neural firing patterns related to epileptic activity, which can be used to locate epileptic lesions [4,5]. In terms of neuromodulation, cochlear implants help deaf patients restore their hearing. Deep brain stimulations are used to relieve Parkinson’s symptoms, and spinal cord stimulators help relieve neuropathic pain [6].

The ideal neural recording and stimulation electrodes should have good biocompatibility, low impedance, and high charge injection capacity for the high-quality, low-damage, and long-term stable neural recording and regulation [7]. Soft neural tissues usually exhibit anisotropic and viscoelastic characteristics that allow enduring the strain and displacement caused by the blood flow, respiration, and body movement. Their mechanical properties are quantitatively described by Young’s modulus. Young’s modulus of the central nervous system (brain and spinal cord) tissue varies between 100 Pa and 10 kPa [8,9,10]. Traditional neural electrodes fabricated from hard materials, such as metals and semiconductors, exhibit a mechanical mismatch with neural tissues, causing inflammation and glial hyperplasia in the tissues around the electrodes [11,12]. This results in the formation of a glial scar, and the activated tissues cause electrode degradation, material corrosion, insulation failure, and electrode impedance fluctuation, which reduce the long-term stability of neural signal recording [13]. Meanwhile, electrode impedance is strongly correlated with the effective surface area of the electrode. Increasing its value can reduce the impedance and lower the thermal noise amplitude [14]. Under normal circumstances, the geometric surface area of the electrode used for the action potential recording of a single neuron should not exceed 2000 μm^2^ and is usually much lower. A small metal electrode may have a large electrochemical impedance, resulting in a low signal-to-noise ratio. To solve this problem, it is necessary to utilize porous electrode materials or modify the electrode surface to obtain a neural electrode with a larger effective surface area but smaller geometric size [15].

The development of flexible bioelectronic devices provides a new strategy for improving the biocompatibility and long-term stability of neural interfaces [16,17]. The size, shape, and tensile modulus of the electrode materials determine their biocompatibility with biological tissues [18]. For example, the decrease in geometrical size and use of flexible materials can reduce the bending stiffness of the electrode, as well as the structural and mechanical differences between the neural electrodes and nerve tissues. Therefore, many studies have been dedicated to achieving stable monitoring and regulation of neural activities by optimizing the geometric structure of the electronic device or using flexible materials [19,20]. For metallic electrodes and other traditional inorganic materials, carbon materials, conductive polymers, hydrogel polymers, and other soft conductive materials are often used as coating electrodes to improve the electrical performance of the electrode–neural interface [21,22,23]. The flexible coating greatly increases the area of charge transfer, providing safe electrical stimulation to tissues while avoiding undesirable chemical reactions and cell damage. Compared with the modification of traditional electrode materials, the development of graphene-based neural electrodes is a relatively simple and cost-effective method. Owing to its excellent bio and neuroelectronic properties, graphene is an ideal material for future nerve probes. Graphene is a two-dimensional nanomaterial with a single atomic layer composed of sp^2^-hybridized carbon atoms [24]. As an active material for nerve electrodes, graphene has many advantages over other materials that include: (1) high mechanical flexibility that allows close contacts with soft brain tissues and formation of a stable electrode–nerve interface [25]; (2) good electrical conductivity and excellent carrier mobility (up to 100,000 cm^2^·V^−1^·s^−1^), which promote the highly sensitive detection of neuroelectric signals by a graphene field-effect transistor [26]; (3) a single atomic layer thickness and ultra-high specific surface area combined with unique electrical properties, which ensure low electrochemical impedance and high charge injection capabilities of graphene neural electrodes that lay the foundation for the effective electrical stimulation of neural tissues [27]; and (4) high transparency and transmittance of a single-layer graphene (up to 97.3%) [28]. Graphene can be prepared by various methods, including liquid-phase exfoliation, micromechanical exfoliation, oxidative exfoliation, chemical vapor deposition (CVD), and SiC epitaxial growth [29]. Among these methods, CVD produces a large-area single-layer graphene film with excellent flexibility and high transparency, which exhibit few crystal defects and high carrier mobility. These advantages facilitate the application of graphene in optoelectronic devices, especially those containing transparent nerve electrode arrays. Graphene sheets may be obtained by exfoliation, and graphene fiber electrodes with high porosity and roughness are produced from graphene sheet suspensions. Therefore, graphene electrodes can record electrophysiological signals while observing changes in cells and blood vessels under the electrodes with electrophysiological activity, providing a technical means for achieving a better understanding of the brain structure and functions. Owing to their high transparency, graphene neural electrodes can also be combined with other technologies (such as optogenetics) for the optical regulation of neuroelectric activities. Due to the above-mentioned advantages, graphene has a high application potential in basic neuroscience and the treatment of brain diseases. In this literature review, we summarize the recent advances related to the graphene application in neuroelectrophysiological detection, including the development of graphene electrodes, regulation of neural cells, and utilization of graphene electrodes for recording neuroelectric signals both in vitro and in vivo. Future development directions of graphene neural electrodes are also discussed.

## 2. Graphene Electrodes

### 2.1. Flexible Graphene Electrodes

**Figure 1:**
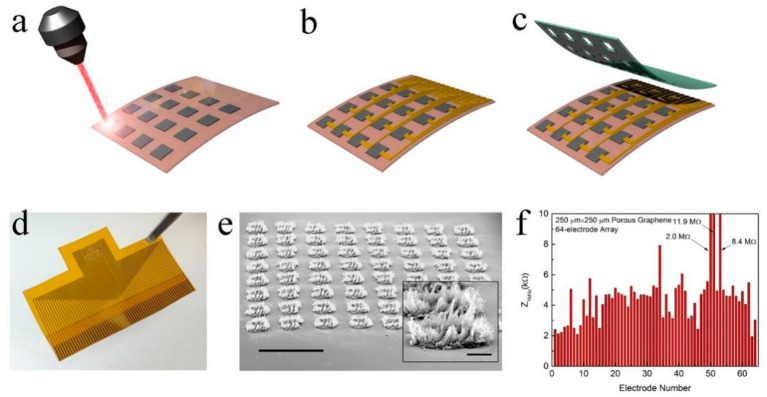
Fabrication of the porous graphene electrode array. Schematics illustrating (**a**) laser pyrolysis, (**b**) metal interconnects, and (**c**) SU-8 encapsulation. (**d**) Photograph of the fabricated 64-electrode array. (**e**) Tilted scanning electron microscopy (SEM) image of the 64-spot porous graphene array. Scale bar: 1 mm. The inset contains the SEM image of an individual spot. Scale bar: 100 μm. (**f**) Impedance of 64 electrodes measured at 1 kHz. Adapted from [7]. Copyright (2016), with permission from Nature Publishing Group.

Graphene is composed of carbon atoms arranged in a hexagonal honeycomb and possesses excellent electrical, mechanical, and chemical properties. The flexibility of graphene enables its high mechanical compatibility with surrounding tissues, which can significantly reduce the damage caused to nerve tissues and help replace silicon and metal materials for novel neural interfaces [30,31]. The porous graphene electrode has a large specific surface area, low impedance, and high charge injection capability, which are conducive to high-quality cortical recording and stimulation [32]. Kuzum and co-workers prepared a three-dimensional (3D) porous graphene foam directly patterned onto a polyimide substrate by laser pyrolysis [7]. After that, they used Cr/Au metal leads and contact pads and negative photoresist SU-8 as an encapsulation layer to obtain a flexible graphene neural electrode array with high porosity and surface roughness (Figure 1). The impedance of this array was approximately two orders of magnitude lower than that of gold electrodes with the same sizes. Chemically doped graphene electrodes were obtained by the treatment with nitric acid, which further reduced the impedance value while increasing the charge injection limit (CIL) from 2 to 3.1 mC·cm^−2^. High CIL is very important for the performance of electrodes since it represents the capability to efficiently deliver charge without exceeding safety limits for both tissue and electrodes. This value is good for application and is superior to other materials such as iridium oxide, carbon nanotube, PEDOT, Ta_2_O_5_, and titanium nitride. Subsequently, the electrodes were placed on the surface of the rat sensory cortex to record sensory evoked potentials. Using an electrode to stimulate the motor cortex can cause flexion of ankle and knee joints. This work could be a powerful tool, especially for electrical microstimulation and mapping spatial-temporal cortical dynamics.

**Figure 2:**
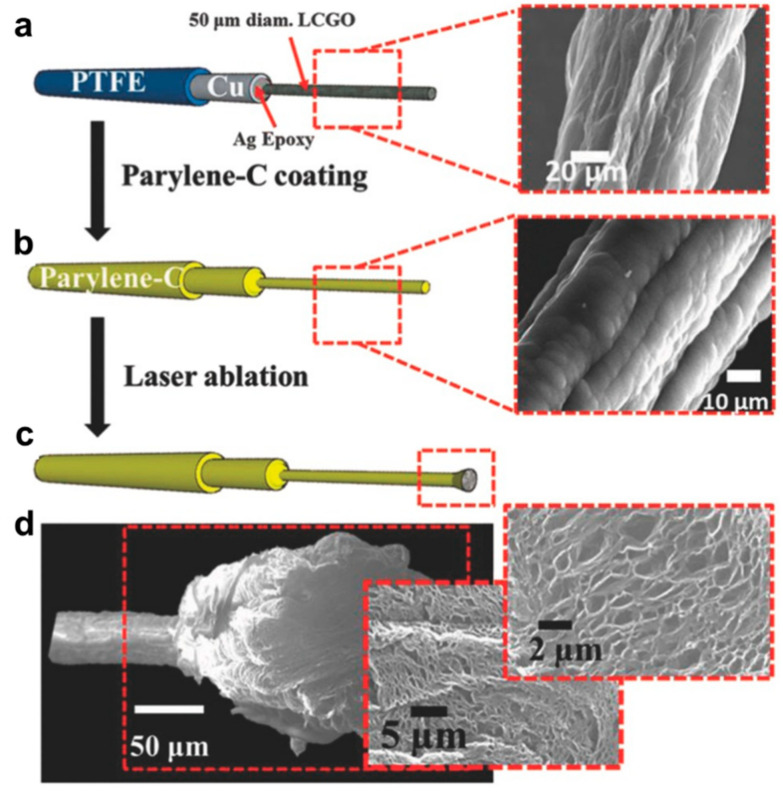
Fabrication and imaging of LCGO brush electrodes. (**a**) The electrodes are attached to polytetrafluoroethylene-insulated copper wires with diameters of approximately 1 mm using conductive silver-based epoxy followed by (**b**) Parylene C coating. (**c**) Laser ablation at 250 mW opens the electrode end, creating a “brush” electrode. (**d**) Laser treatment produces an amorphous electrode with extraordinary surface roughness and porosity. Adapted from [11]. Copyright (2015), with permission from WILEY-VCH Verlag GmbH & Co. KGaA, Weinheim.

Garrett et al. prepared graphene oxide fibers by wet spinning and annealed them at 220 °C to obtain liquid crystal graphene oxide (LCGO) fibers [11]. Parylene C was used as the insulating layer, and the fiber ends were lasered. A neural electrode with high charge injection capability was obtained by the ablation into a brush to increase the surface roughness and nano-porosity (Figure 2). The researchers further used this electrode to stimulate ganglion cells in the detached rat retina in vitro and simultaneously performed whole-cell patch clamp recording. Afterwards, water-soluble sucrose was coated onto the electrode surface to form microneedles, and the flexible electrode was implanted into the cat’s visual cortex. Finally, the sucrose layer was dissolved, and the neural activity was recorded. The highlight of this work is the development of a freestanding and flexible shank seamlessly attached to the electrode. This could save the trouble of fabricating interfaces between mismatched materials or welding a larger electrode onto the end of a smaller wire.

Graphene can also be integrated with other materials to prepare nerve electrodes, thus combining the advantages of multiple materials. Jang et al. prepared a neural probe using a (poly(3,4-ethylenedioxythiophene, PEDOT)-Au-ZnO nanowire composite material at the recording site and Au-graphene lead wire [33]. The combination of ZnO nanowires with the conductive polymer PEDOT coating considerably increased the effective surface area and charge storage capacity of the electrode and reduced its impedance. To compare the performance characteristics of Au and Au-graphene, the electrode leads were repeatedly folded 30 times. It was found that the Au lead exhibited significant increase in electrode impedance, whereas the impedance of the Au-graphene electrode lead changed very little after 100 cycles of repeated folding. These results indicated that graphene addition increased the electrode bending resistance, while the Au-graphene combination ensured high flexibility and conductivity of the electrode. At the same time, this electrode successfully recorded local field potential (LFP) signals under beard stimulation.

### 2.2. Transparent Graphene Electrodes

Graphene is used in many research fields due to its excellent electrical conductivity, thermal conductivity, transferability, and mechanical strength. Owing to the high light transmittance over a wide spectral range, graphene can be utilized in transparent neural electrode arrays to realize the multifunctional integration of electrophysiological recording, optical imaging and optogenetic stimulation, which may help study neural circuits with high time-spatial resolution [34].

Graphene-based transparent electrode arrays have a large transmittance area of incident light, which can directly illuminate the neurons below the site. In contrast, for opaque metal electrodes, only the area around the electrode can be stimulated, which negatively affects the results of high-resolution optogenetic experiments.

**Figure 3:**
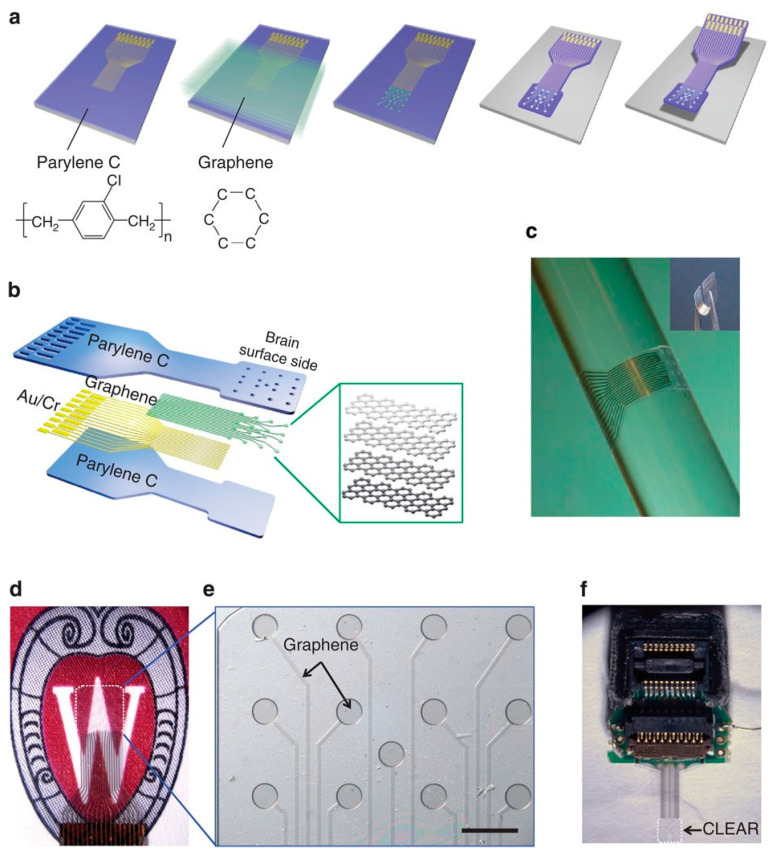
CLEAR micro-ECoG device. (**a**) Basic fabrication process: metal patterning of traces and connection pads on the Parylene C/silicon wafer substrate. Transfer and sequential stacking of four graphene monolayers. Graphene patterning to produce electrode sites. Second Parylene C deposition and patterning to assemble the main device components. Removal of the produced device from the silicon wafer. (**b**) Diagram of the CLEAR device construction displaying the layered structures. (**c**) Demonstration of the CLEAR device flexibility by wrapping a glass bar with a radius of 2.9 mm. (**d**) Rat brain-sized CLEAR device with an electrode area of 3.1 × 3.1 mm^2^ outlined by the white dashed line. (**e**) Magnified image of the rat-sized CLEAR device showing transparent graphene electrode sites and traces on the Parylene C substrate. The displayed side touches the brain surface. Scale bar: 500 mm. (**f**) Mouse brain-sized CLEAR device with a zero-insertion force printed circuit board connector (electrode area: 1.9 × 1.9 mm^2^). Adapted from [35]. Copyright (2014), with permission from Nature Publishing Group.

Williams et al. prepared a carbon-layered electrode array (CLEAR) based on four-layer graphene [35]. This device contained 16 graphene electrode sites, and Parylene C was used as the electrode substrate and encapsulation layer (Figure 3). Its transmittance in the ultraviolet to infrared region exceeded 90%. The amplitude of the optical artifacts generated during illumination depended on the optical power and duration of the optical stimulation. Therefore, reducing the optical power could decrease or completely eliminate these artifacts. The CLEAR device was placed in the cerebral cortex of a Thy1::ChR2 transgenic mouse, and a neuroelectrical signal was recorded under the optogenetic stimulation with 473 nm blue light. At the electrode recording site, the fabricated CLEAR device successfully performed the fluorescent imaging and optical coherence tomography of cortical blood vessels due to the wide spectrum of graphene light transmission. Moreover, the transparent electrode recording site did not block the underlying tissue that was clearly imaged.

In the subsequent work, the research used transparent graphene microelectrode arrays to perform micro electrocorticography (μECoG) studies, and the simultaneous neuroelectrical stimulation and neural activity imaging of the cortex of transgenic GCaMP6f mice [36]. The light transmittance of graphene allowed the neural activity induced by electrical stimulation to be visualized by fluorescent calcium imaging. They found that the CIL of the graphene electrode was as high as 116.07–174.10 μC·cm^−2^. In addition, the use of cathodic stimulation induces a stronger neural response than anode stimulation, confirming that the charge is more effectively transferred to the brain. These studies demonstrate the advantages of the highly light-transmitting neural electrode arrays over non-transparent metal electrodes in electrophysiological applications, optical imaging, and optogenetic experiments.

Kuzum et al. used transparent flexible graphene neural electrode arrays to perform simultaneous optical imaging and electrophysiological recording [13]. The electrode contained a polyimide flexible substrate, a p-type doped graphene site, and an SU-8 encapsulation layer. The doped graphene electrode exhibited a low impedance and high charge storage capacity. It could simultaneously perform calcium ion imaging and electrophysiological recordings of hippocampal tissue slices without introducing optical artifacts. Furthermore, transparent graphene electrodes detected high-frequency electrical activity, which complemented calcium imaging with high spatial resolution but low temporal resolution. Wrapping graphene around the Ag electrode significantly inhibited its corrosion. After immersing the graphene-coated Au electrode into a phosphate buffer for six months, its Raman spectrum contained the characteristic graphene peaks, which indicated that graphene could be used not only for fabricating transparent electrodes with low noise levels, but also as a corrosion protection layer for metal microelectrodes with long-term stability.

Thunemann et al. applied an electrochemical bubbling method to transfer graphene onto a 50-μm thick polyethylene terephthalate substrate [37]. The graphene layer was patterned into electrode sites, and its surface was cleaned to avoid the formation of cracks and organic matter residue. Finally, using the SU-8 encapsulation layer, a transparent graphene microelectrode array with 16 channels was obtained. The impedance of the electrode was less than 1.5 MΩ (1 kHz), and it was repeatedly bent 20 times with a radius of curvature of 5 mm (which was within the natural bending range of the mouse cortex) without failure. After that, the electrode was placed on the surface of the mouse primary somatosensory cortex to perform the two-photon imaging of interneurons and blood vessels from the cortex surface to a depth of 1200 μm. Note that the same electrode can be used to stimulate the LFP and calcium ions of the contralateral beard with a single pulse. This includes the synchronous recording of ion transient signals, LFP signal recording under optogenetic control, two-photon imaging of arteriole expansion, and simultaneous hemodynamic optical imaging, and neuroelectric activity recording under beard stimulation.

**Figure 4:**
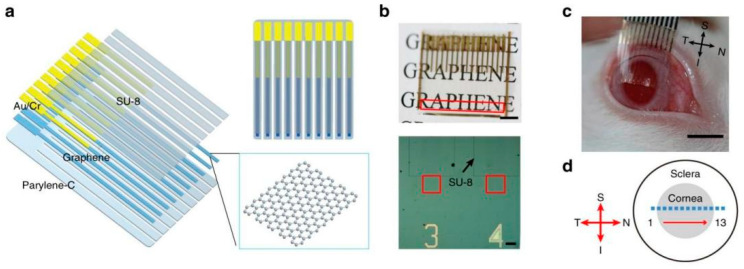
Multi-electrode ERG recording with the soft transparent graphene electrode array. (**a**) Diagram of the graphene multi-electrode array construction, showing the layered structures. (**b**) Top: a soft transparent graphene electrode array positioned over a piece of printed paper to demonstrate its optical transparency. Scale bar: 5 mm. The recording sites arranged in a linear pattern are located in the region marked by the red box. Under each recording site, there is an optical channel patterned with Au. Bottom: optical microscopy image showing some graphene electrode sites and traces. The red box marks the graphene recording sites. The black arrow points to the patterned SU-8 insulation layer on one electrode. Scale bar: 150 μm. (**c**) A stripped graphene electrode array positioned over a dilated rabbit eye. Scale bar: 5 mm. (**d**) A schematic drawing showing the positions of the recording channels (marked by the squares) on a rabbit eye. Channels 1–13 are evenly distributed over the cornea equator spanning from the temporal area to the nasal periphery. Adapted from [38]. Copyright (2018), with permission from Nature Publishing Group.

Transparent electrodes can also be utilized in electroretinography (ERG) studies. Duan et al. prepared flexible and transparent graphene contact lens electrodes (GRACEs) (Figure 4a) [38] with excellent light transmittance and low impedance over a wide spectral range that formed a conformal tight interface with the cornea. During conventional ERG recording, wearing this electrode did not cause any visible damage to the cornea. Using this electrode, the researchers achieved high-quality recordings of a variety of ERG signals. In the full-field ERG region, GREACEs can record higher corneal potential amplitudes than those obtained by the commercial ERG-Jet electrodes. These electrodes may also be used to record multifocal ERG signals (Figure 4b) due to the preservation of the refractive power of the eye by the conformal interface. In addition, the multi-site transparent graphene electrode array (Figure 4c,d) was employed for detecting the spatially resolved ERG response. It was found that the ERG signal amplitude was highest in the cornea center and decreased in the temporal and nasal areas.

## 3. Interactions between Graphene and Neural Cells and the Detection of Neuroelectric Signals In Vitro

Neural cells are an important part of the brain tissue, and the generation of neuroelectric signals is mainly based on the electrophysiological activity of neural cells. During such activity, large amounts of potassium and sodium ions pass through ion channels to change the potential of the extracellular environment [39]. Neural electrodes can record changes in the extracellular potential to help better understand the neural activity and the related pathological mechanism. Using neural electrodes to monitor the electrical signals of cells or tissues in vitro, it is possible to avoid invasive damage to the living body and provide guidance for in vivo studies.

### 3.1. Interactions between Graphene and Neural Cells

Graphene exhibits high stability and biocompatibility; therefore, it can serve as an ideal platform for the cultivation of neural cells. In addition, graphene may regulate the growth, differentiation, and proliferation of cells cultured on its surface [40]. This enhances the electrical performance of the neural network, thereby helping to rebuild the damaged central nervous system and accomplish neural repair. Graphene has a hydrophobic structure, and its modification by hydrophilic molecules such as polyglycol, polylysine, hyaluronic acid, and polyacrylic acid can promote the adhesion of cells to the graphene surface, leading to the survival and growth of neural cells [17,41,42,43,44]. For example, Cheng et al. used graphene films prepared by chemical vapor deposition as the culture substrate and coated a polylysine layer on its surface to enhance the adhesion of cells [45]. The cultured mouse hippocampal neurons exhibited faster synaptic germination and growth in early culture compared to the traditional polystyrene substrate. In addition, Chen et al. cultured rat adrenal pheochromocytoma (PC12) cells, oligodendrocytes, and osteoblast cells on single-walled carbon nanotube networks and graphene films [46]. The obtained results revealed that all three cell types multiplied well on the graphene films. However, the cell proliferation and activity on the carbon nanotube networks were inhibited.

**Figure 5:**
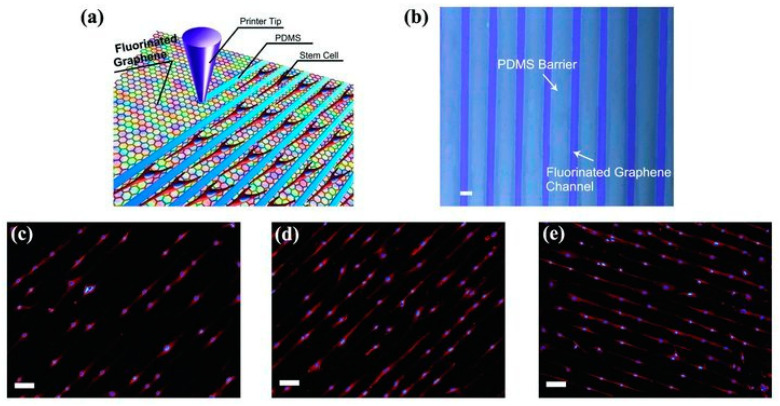
(**a**) Schematic illustration of patterning MSCs by directly printing polydimethylsiloxane (PDMS) barriers on graphene films. (**b**) Optical microscopy image of the printed PDMS pattern on a fluorinated graphene film (scale bar: 50 μm). (**c**–**e**) Aligned growth of stem cells on graphene, partially fluorinated graphene, and fluorinated graphene with a printed PDMS pattern, respectively (scale bar: 100 μm). Adapted from [48]. Copyright (2012), with permission from WILEY-VCH Verlag GmbH & Co. KGaA, Weinheim.

In recent years, neural stem cells have attracted significant attention from researchers as a group of self-renewing cells in the central nervous system. Neural stem cells have two main characteristics: (1) unlimited self-renewing ability and (2) versatility, which can be differentiated into all types of cells in the central nervous system. Using neural stem cells for brain repair and nerve regeneration is critical for the effective treatment of various neurological diseases. The differentiation of neurostem cells is simultaneously influenced by extracellular and intracellular factors, particularly their specific microenvironment and metabolic state. Bietic microenvironments can be constructed by modifying the surfaces of graphene and graphene oxide with collagen, laminin, and broninin. This promotes the attachment, growth, proliferation, and differentiation of neurostem cells. Park et al. used a laminin-modified graphene membrane as a substrate to cultivate human neurostem cells and found that graphene substrates could provide a more favorable micro-environment for human neural stem cells, thus dividing them into neurons [47]. Moreover, under a chemical, physical, or mechanical stimulation, graphene may promote the differentiation of human mesenchymal stem cells (MSCs) into tissues of non-mesenchymal sources, such as neurons. Loh et al. utilized the strong polarity of carbon–fluorine bonds in graphene fluoride to induce the differentiation of bone marrow MSCs into a neuron spectrum (Figure 5) [48]. They found that the highly polarized graphene changed the cell morphology from irregular polygons to a shuttle with simultaneously growing nuclei, which was more favorable for neuron differentiation. Furthermore, in the absence of chemical inductors, the differentiation of MSCs into a neuron lineage can also be induced by graphically rendering graphene fluoride into directionally aligned long microchannels.

Although cell adhesion to the graphene surface can be enhanced by modifying it with amino acids and proteins, these coatings also increase the impedance of neurons and the electron–nerve interface. The latter affects the charge transfer at the interface and reduces the sensitivity and reliability of electrophysiological signal detection. Therefore, the cultivation of neural cells directly on the graphene substrate can enhance the contact and electrical coupling of graphene and neural cells, thus helping detect weak signals. Picaud et al. reported for the first time that primary retinal ganglia cells could survive on a graphene substrate without any glial support layer or protein coating, although its synaptic length was lower than that of a polylysine-coated graphene substrate [49]. Prato cultured hippocampal neurons directly with micrometer-sized graphene sheets prepared by liquid-phase stripping and spherical grinding. The graphene substrate was found to be an inert neural interface material that preserved the electrophysiological properties of neural cells and did not affect their charge transfer process [50]. Furthermore, Delacour et al. found that graphene crystallinity played an important role in the attachment, growth, and axial formation of neural cells. Therefore, high-quality graphene is required for the effective adhesion of neural cells on its surface, as the decrease in graphene crystallinity changes the graphene surface from highly adhesive to completely exclusive towards these cells [51].

**Figure 6:**
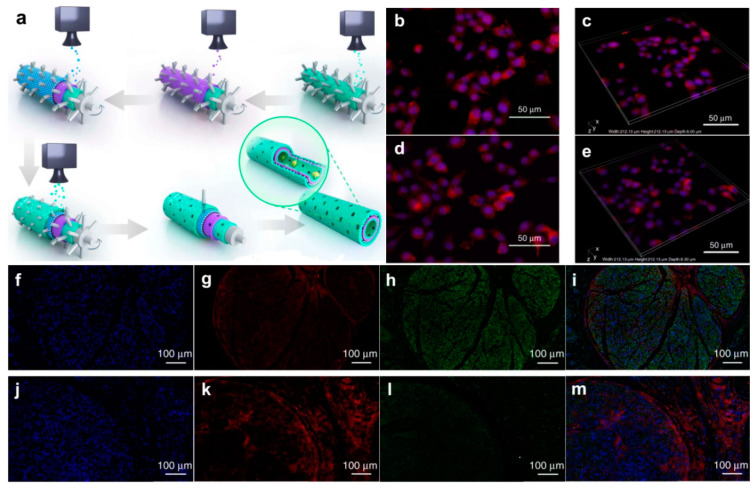
Fabrication of graphene nerve conduit with LBLC method and its application in axonal regrowth and remyelination. (**a**) The inner-most and outer-most green layers are PDA/RGD mixed layers. The purple layer is single-layered or multi-layered graphene and PCL mixed layer. The blue layer is a repetition of the graphene and PCL mixed layer. (**b**–**e**) Immunofluorescent staining for Ki67 and F-actin. (**b**,**c**) Ki67 expression of SC on PDA/RGD-SG/PCL. (**d**,**e)** Ki67 expression of SC on PDA/RGD-MG/PCL. (**f**–**m**) Triple immunofluorescent staining of Tuj1 and NF200 at 18 weeks post operatively. Tuj1 (green), NF200 (red), and nuclei (blue) were exhibited from different groups, respectively. (**f**–**i)** SC-loaded PDA/RGD-SG/PCL. (**j**–**m**) SC-loaded PDA/RGD-MG/PCL. Adapted from [55]. Copyright (2018), with permission from Nature Publishing Group.

Neural cells in organisms exist in 3D environments. Three-dimensional graphene scaffolds can better simulate the real environments of organisms, such as cell–cell and cell–matrix interactions, than can graphene films [52]. Therefore, 3D graphene scaffolds used as cell culture substrates more accurately reflect the electrophysiological behavior of neural cells in living organisms. In addition, these scaffolds promote the proliferation and differentiation of PC-12 cells and neural stem cells [53]. The unique nanoscale porous structure of 3D graphene foam and reduced graphene oxide fiber cell scaffolds ensures the large-scale transport of nutrients required for the metabolism of neural stem cells, resulting in active cell proliferation and differentiation. Cheng et al. used 3D graphene foam synthesized by CVD on Ni foam to cultivate human neural stem cells. It was found that the produced foam significantly enhanced the differentiation of human neural stem cells to neural and glial cells [54]. Due to their high conductivity, graphene electrodes can also be used to stimulate differentiated human neural stem cells. In addition, 3D tubular graphene scaffolds form cell structures with highly accurate geometries. Brain-like functional tissues may be constructed by the higher-order assembly of spatially arranged cell structures. Yuan et al. further developed an integrated layer-by-layer casting (LBLC) method to apply a 3D graphene substrate to an artificial neural catheter containing printed polydopamine/arginylglycylaspartic acid and graphene/polycaprolactone alternating layers (Figure 6) [55]. Polydopamine and polypeptide increased the cell affinity to the graphene surface, while graphene and polycaprolactone strengthened the tubular structure and increased its rigidity for the long-term physical studies. This neural catheter effectively promoted the regeneration of axons and myelin in the animal models of sciatic neural damage, resulting in peripheral nerve repair.

Neural network electrical signals reflect the electrophysiological synergistic activity of group neurons that form functional connections. Studying the electrophysiological activity of neuron networks is a prerequisite for achieving a better understanding of the neural loop formation. The reasonable regulation of neural network electrical signals is highly important for the successful treatment of neurological diseases. Cheng et al. studied the differentiation of human neural stem cells on a graphene substrate and found that the differentiated cells grew on the graphene surface and formed functionally connected networks [56]. Furthermore, the excitability and activity of the neural network on the graphene substrate were significantly enhanced, and the feasibility of graphene application for regulating the neural network behavior in vitro was demonstrated. Later, Scaini et al. reported that monolayer graphene limited the mobility of potassium ions in the vicinity of its surface to regulate neural communication and increase the ion current [8]. It was shown that the neuronal synaptic current recorded on the graphene substrate had a significantly higher frequency than that of a control group. These results indicate that graphene exhibits good compatibility with neural cells. Furthermore, graphene can effectively regulate neural cell growth behavior and neural network electrical signals. Based on these characteristics, graphene can be used to develop a friendly neural interface for the preparation of new neural electrodes.

### 3.2. Graphene Neural Electrodes for In Vitro Detection of Neuroelectric Signals

**Figure 7:**
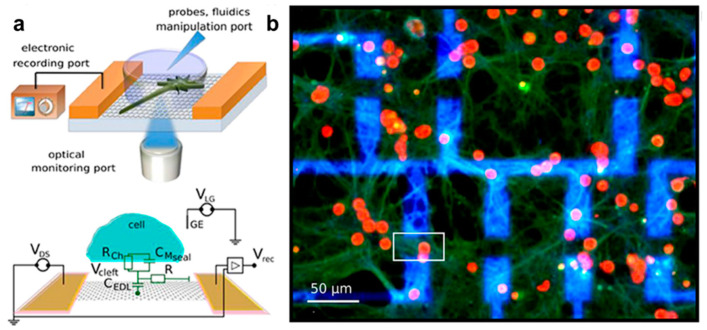
Graphene microelectrodes for the in vitro neural activity recording. (**a**) Schematicillustration of the inverted microscope setup containing transparent graphene electrodes. (**b**) Immuno-fluorescence micrographs of the neurons cultured on the graphene field effect transistors. Adapted from [58]. Copyright (2017), with permission from Frontiers.

Graphene has superior biocompatibility and electrical properties for the effective functional interaction with neural tissues, enabling a wide range, high temporal resolution, long-term stable regulation, and recording of neuro electrophysiological activity. Moreover, unlike the conventional metal electrodes, graphene can be transferred to a transparent substrate to prepare transparent neural electrode arrays while recording electrophysiological signals without negatively affecting the imaging quality. Graphene-based liquid gate transistors and electrode arrays have been widely used for the extracellular action potential measurements of cardiac cells. The electrophysiological activity of the cortical neural network is more complex and random than that of cardiomyocytes and myocardial-like cells. Therefore, enhanced electrode performance is required in the former case. To detect and record the electrophysiological signals of a single neural cell, the size of graphene microelectrodes should be close to the size of an individual neuron. However, as the microelectrode size decreases, the impedance increases. As a result, the thermal noise of the device increases, and the signal-to-noise ratio decreases. Compared with microelectrode arrays, transistor devices can perform signal self-amplification and are characterized by low noise and high sensitivity levels. Moreover, their transconductance depends on the channel geometry, breaking through the limitations of the microelectrode array size effects and making it easier to build neural electrodes with high spatial resolutions. Xu et al. recorded, for the first time, the self-distributed electrical signals of cortical neurons cultured on their surface using a graphene liquid gate transistor electrode with superior cross-conductivity properties and lower noise levels than those of similar transistor devices [57]. In addition, the fabricated device exhibited high mechanical flexibility, and after repeated bending, its performance remained unchanged, laying the foundation for the preparation of flexible neural electrodes for the detection of live electrophysiological signals. Thereafter, Veliev et al. prepared a graphene liquid gate transistors on flexible polyimide and transparent substrates and used them to record the mouse hippocampal neuron signal (Figure 7) [58]. The obtained results indicated that the graphene liquid gate transistors had the highest sensitivity, which might be related to the lower charge density at the sapphire substrate interface.

To construct a more stable graphene–neuron interface, Offenhausser et al. developed a new graphene liquid gate transistor packaging method [59]. By packaging only the metal wire on the chip, cultured neurons avoid the bending stress generated near the graphene surface, thus reducing the gap between neurons and graphene. Because the graphene layer has a single atom thickness, it must be often transferred from the growth substrate to the device substrate. This process introduces polymer residues onto the graphene surface, thus increasing the contact resistance between graphene and the metal electrode, causing a high contact noise level. Garrido and co-workers used ultraviolet ozone to remove the residue from the graphene surface and reduce the contact resistance between the graphene and metal components [60]. They found that the ozone-treated graphene devices possessed lower contact noise levels and higher signal-to-noise ratios than those of the untreated devices. These results indicate that graphene exhibits excellent electrical performance and biocompatibility during in vitro neural signal recording. Through surface modification or hybridization, the impedance of graphene and the biological interface can be further reduced, thus increasing the signal-to-noise ratio of the recorded electrophysiological signal and laying the foundation for its recording in vivo.

## 4. Graphene Neural Electrodes for In Vivo Detection of Neuroelectric Signals

The detection of neurophysiological signals in vivo is less hindered by the external culture environment and reflects real neural activity. Graphene can be utilized for long-term stable recording and electrical stimulation without causing serious immune reactions due to its excellent mechanical flexibility, biocompatibility, and electrical properties. This section describes the application of graphene as a material for intracranial (both cortical and implantable) electrodes.

### 4.1. Graphene-Based Cortical Electrodes

The cortical electrode can record the integrated signals of multiple neurons while causing less trauma to the tissue than the implanted electrodes. It has been widely used in the clinical diagnosis of neurological diseases, such as epilepsy. Graphene exhibits good transparency and excellent conductivity properties and thus may be employed for constructing transparent cortical electrodes. This combination of neural electrodes with optical imaging technology can simultaneously perform tissue observation and neural signal detection, contributing to a clearer and more complete understanding of the structure and functioning of the brain.

Although chemical treatment (including plasma treatment and nitrate doping) can reduce graphene impedance, its effect is limited due to the increase in the hole concentration caused by the decrease in the Fermi level of graphene. However, because the quantum capacitance of graphene is very small, the density of states of the Dirac dot is also low. Therefore, the reduction in the Fermi level increases the hole concentration very little. To solve this problem, Kuzum et al. developed a method for reducing the electrode impedance by the electrodeposition of platinum nanoparticles on the graphene surface [61]. The obtained electrodes were able to exceed the quantum capacitance limit, and their impedance decreased by a factor of 100 while maintaining a high transparency level. The produced graphene/Pt nanoparticle microelectrode array enabled the calcium ion imaging of cells at various depths while recording surface neural signals in the cerebral cortex. In addition to doping and surface modification, graphene electrode impedance can be effectively reduced by varying the graphene morphology.

**Figure 8:**
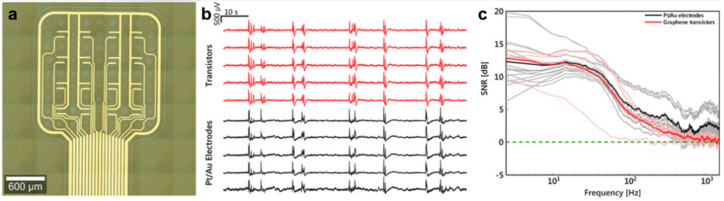
**Characteristics of single-graphene field effect transistor** (**SGFET**) **arrays**. (**a**) Optical microscopy image of the active area of a 4 × 4 graphene SGFET array. (**b**) Spontaneous oscillatory activity. (**c**) Signal-to-noise ratio versus frequency curves extracted from the PSD for graphene SGFETs (light red lines) and platinum black electrodes (gray lines). The mean values are depicted by the bold lines. Adapted from [62]. Copyright (2018), with permission from WILEY-VCH Verlag GmbH & Co. KGaA, Weinheim.

Electrode impedance is not the only factor determining the electrode performance, because thermal noise is not the only type of noise affecting the neural recording process. Electronic noise, biological noise, and other noises related to the recording system negatively affect the detection and classification of neural electrical signals. Unlike microelectrode arrays, field effect transistors possess intrinsic signal amplification properties and may effectively reduce the external noise, thus increasing device sensitivity. A graphene liquid gate transistor was prepared by Garrido and co-workers from single-layer graphene grown by CVD on a polyimide substrate [62]. Using this transistor, spontaneous slow-wave activity in the cerebral cortex, response signals from the sono-acoustic cortex, and synchronous discharge activity of epilepsy were recorded for the first time. The authors found that the signal-to-noise ratio of the graphene electrode in the low-frequency range was higher than that of a platinum black electrode (Figure 8).

Afterwards, they conducted a more detailed study, in which the graphene liquid gate transistor was used for recording cortical signals. The obtained results confirmed that this transistor could detect neural signals in a wide electrophysiological bandwidth. In particular, its high signal-to-noise ratio in the low-frequency range was suitable for recording cortical diffusive suppression signals, enabling the detection of low-frequency signals with high reliability and spatial resolution [63]. Furthermore, to avoid the split declination caused by the large contact resistance between the metallic source drain and the graphene channel and minimize the mismatch of Young’s modulus between graphene and the contact electrode during flexible deformation, Fang et al. developed a full-carbon graphene transistor consisting of graphene channels and a source drain fabricated from a graphene/carbon nanotube hybrid film [64]. Because graphene in the channel is formed synchronously with graphene in the hybrid electrode, a seamless connection structure is produced between the transistor channel and the source drain electrode. In addition, compared to the conventional metal electrodes, the graphene/carbon nanotube hybrid electrode is more mechanically compatible with the graphene channel. For this reason, the transconductance of graphene does not change when the device undergoes bending deformation. The same researchers prepared all-carbon fold nano-neuroelectrodes by mechanical compression [65]. Compared with a planar electrode, these electrodes exhibited a lower projection area at the same effective area; as a result, the device possessed a higher spatial resolution than that of the planar device during the detection of epileptic signals in rats.

As an effective tool for detecting brain activity, cortical electrodes can reveal the roles of specific brain areas in body functions. However, some specific information (such as precise movement) is transmitted by a small number of neurons in the form of a single potential peak. The electrophysiological signals of such weak neural populations easily disappear in the cerebral cortex and, therefore, cannot be detected, which also limits the application of cerebral cortex electrodes.

### 4.2. Detection of Neuroelectric Signals Graphene-Based Implantable Electrodes

Compared with cortical neural electrodes, implantable electrodes form better contacts with neurons, thereby detecting the electrophysiological signals of weak neural clusters. The electrical signal of a single neuron can be recorded by reducing the size of the electrode detection site. Because graphene possesses good biocompatibility and electrical properties, it is utilized to coat the surface of rigid nerve electrodes and reduce the immune response of brain tissues to neural electrodes.

Delacour et al. coated a single-layer graphene film on the Michigan electrode surface [66]. The obtained results revealed that the proliferation of astrocytes and microglia around the electrode was reduced while the signal quality and stability were increased after the coating procedure. Duan et al. applied seamless, full-coverage graphene coatings on copper microwires by low-pressure chemical vapor deposition, which effectively reduced their cytotoxicity [67]. The fabricated electrodes can be used to record local field potentials and single-cell action potentials in the rat brain without producing image artifacts in the magnetic resonance imaging (MRI) scanner. However, the instability of the coating–electron combination may cause coating delamination and related electrode failure during the long-term use. To prevent the coating falling off, Garrett et al. prepared soft reduced graphene oxide microfibers by wet spinning and employed them as independent neural stimulation and recording electrodes [11]. They used Parylene C to encapsulate and reduce graphene oxide microfibers and laser-ablated the front end to form a “brush”-like electrode with an enhanced neural interface. Subsequently, Wallace et al. fabricated a low-impedance graphene composite microelectrode by modifying the Pt coating on graphene fibers [68]. The results of in vivo studies revealed that the electrodes implanted into the rat cortex could detect neuronal activity with a high signal-to-noise ratio in areas as small as that of a single neuron. Similarly, graphene transistors may also be used as implantable neuroelectrodes for neuroelectric signal recording (Table 1).

## 5. Conclusions

Graphene possesses many unique characteristics that allow its potential use as a neural electrode material. They include high biocompatibility, chemical stability, flexibility, optical transparency, and electrical conductivity, which facilitate the construction of a two-way neural interface for the simultaneous detection and regulation. This literature review systematically discusses various types of graphene electrodes, interactions between graphene and neural cells, and possible applications of graphene microelectrode arrays and transistors in the detection and stimulation of the neurophysiological signals of neural cells, brain tissue slices, and living brains. Recently conducted research studies revealed that graphene was highly compatible with neural cells and promoted the differentiation of neural stem cells into neurons. Furthermore, it also enhanced the electrical signals of the neural network. The flexible neural electrode prepared by transferring a graphene layer onto a flexible substrate was better attached to the brain tissue than traditional rigid metal electrodes and achieved high temporal and spatial resolutions and signal-to-noise ratio of the neural electrical signal. In addition, the graphene-based flexible transparent neural electrode exhibited high mechanical flexibility and transparency, indicating that it could simultaneously perform optical imaging and electrophysiological signal recording as well as the optogenetic regulation of the activity of neural cells under the electrode. Finally, graphene implantable electrodes may effectively reduce the immune response of the brain tissue to increase the durability of neural electrodes.

Although graphene provides new possibilities for the construction of ideal neural electrodes, graphene-based neural electrodes can be further improved in the following ways. (1) To achieve higher signal-to-noise ratio as well as time and spatial resolutions and to perform high-density, high-throughput integration of flexible electrodes, appropriate surface treatment methods, graphene pore structure design, and multiplexing and wireless transmission technologies must be developed. (2) To analyze the neural loop mechanism, it is necessary to determine the application potential of graphene materials in implantable neural electrode detection techniques. (3) The new generation of neural electrodes must simultaneously provide multiple functions, including electrophysiological signal recording and regulation, neurotransmitters and other neural-related biomolecule recognition techniques, and the ability to effectively control drug delivery. (4) The neural electrode technique should be combined with other brain regulation and detection tools (such optogenetic technology and MRI), which facilitate studying brain processes. Thus, a multifunctional composite graphene electrode with ultrahigh sensitivity and stability can potentially be utilized in basic neuroscience and for the treatment of brain diseases. This should motivate researchers to continue their detailed studies in this cutting-edge interdisciplinary field.

## Figures and Tables

**Table 1:** Graphene-based electrode materials for neural activity detection.

Materials	Applications	Ref.
Porous graphene electrode array	Cortical microstimulation and sensing; in vivo	[7]
Liquid crystal graphene oxide (LCGO) fibers	Neural stimulation and recording electrodes;	[11]
Graphene-based flexible electrode array with Au-ZnO-Au-PEDOT	Neural stimulation and recording electrodes; in vivo	[29]
Graphene-based carbon-layered electrode array	Neural stimulation and recording Electrodes, optogenetic stimulation, fluorescence, and OCT imaging; in vivo	[31]
Transparent graphene microelectrocorticography (μECoG) electrode arrays	Electrical neural stimulation and simultaneous, fluorescence imaging; in vivo	[32]
Transparent graphene microelectrodes on flexible polyimide (Kapton) substrates	Electrophysiological recording and optical imaging; in vivo	[13]
Crack- and residue-free graphene microelectrode array	2-photon imaging, simultaneous electrical recording, 2P Ca^2+^ imaging, optogenetics, and hemodynamic imaging; in vivo	[33]
Soft graphene contact lens electrodes (GRACEs)	Conformal full-cornea recording of electroretinogram; in vivo	[34]
Monolayer graphene with hyaluronic acid (HA)-based coating	neurons adhesion, neuritogenesis, and intracortical probe; in vivo	[17]
Laminin-coated graphene film	Enhanced differentiation of human neural stem cells into neurons; in vitro	[42]
Fluorinated graphene sheets	Promoting neuro-induction of stem cells; in vitro	[43]
Micrometer-sized graphene sheets prepared by liquid-phase stripping and spherical grinding	Hippocampal neurons culturing of stem cells; in vitro	[45]
3D graphene foams	NSC differentiation and proliferation of stem cells; in vitro	[49]
Multi-Layer 3D PDA/RGD coated graphene loaded PCL nanoscaffold	Peripheral nerve restoration; in vivo	[50]
Solution-gated graphene field effect transistor	Neural activity recording, bioelectronic measurements; in vitro	[52,53,54,57]
Wafer-processed graphene solution-gated field-effect transistors	Neural signal recording; in vitro	[55]
Platinum nanoparticles (PtNPs)/graphene electrodes	Multimodal monitoring of cortical potentials and cellular activity	[56]
Carbon nanotube network embroidered graphene films	Mapping of cardiac signals; in vivo	[59]
All-carbon transistor with a graphene channel and hybrid graphene/CNT electrodes	Brain activity recording; in vivo	[60]
Continuous monolayer graphene coating	Intracortical probes for long-lasting neural activity monitoring; in vivo	[61]
Graphene encapsulated copper microwires	MRI compatible implantable neural electrodes; in vivo	[62]
Graphene-fiber (GF)-based microelectrode arrays with a thin platinum coating	Neural stimulation and recording; in vivo	[63]

## Data Availability

The data supporting the findings of this study are available within the article.
